# *Arthrospira platensis* and *Chlorella vulgaris* Consumption on Iron Status: A Systematic Review of In Vivo Studies

**DOI:** 10.1002/mnfr.70318

**Published:** 2025-11-19

**Authors:** Alexandra Lacurezeanu, Dan Cristian Vodnar

**Affiliations:** ^1^ Faculty of Food Science and Technology University of Agricultural Sciences and Veterinary Medicine Cluj–Napoca Romania; ^2^ Institute of Life Sciences University of Agricultural Sciences and Veterinary Medicine Cluj–Napoca Romania

**Keywords:** anemia, hematology, hemoglobin, iron deficiency anemia, microalgae

## Abstract

Iron deficiency anemia (IDA) affects over two billion people globally and is often treated with conventional iron supplements, which frequently have poor tolerability and limited bioavailability. This systematic review examines the potential of *Arthrospira platensis* (Spirulina) and *Chlorella vulgaris* as alternative, bioavailable iron sources. A systematic search was conducted by Preferred Reporting Items for Systematic Reviews and Meta‐Analyses (PRISMA) guidelines, identifying 32 in vivo studies (7 human, 25 animal) that evaluated iron‐related outcomes of microalgae supplementation. Both *A. platensis* and *C. vulgaris* improved hematological parameters, including hemoglobin, serum ferritin, and red blood cell counts. *A. platensis* showed more vigorous erythropoietic activity, while *C. vulgaris* enhanced antioxidant defenses, increasing superoxide dismutase (SOD) and glutathione peroxidase (GPx) activity and reducing lipid peroxidation. Both microalgae reduced inflammation‐induced hepcidin levels, thereby supporting improved iron absorption. No significant adverse effects or organ toxicity were reported in any of the included studies. *A. platensis* and *C. vulgaris* are safe and effective microalgal supplements that enhance iron status and antioxidant defense, presenting promising alternatives to conventional iron therapy. However, longer‐term human clinical trials are needed to validate these findings and determine optimal dosing strategies.

## Graphical abstract

This graphical abstract provides a comparative overview of the mechanisms by which *Arthrospira platensis* and *Chlorella vulgaris* exert their effects. It highlights significant differences in their biological pathways, molecular interactions, and physiological effects, providing a clear visual summary.

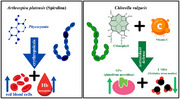

**Abbreviations**
DMT1
: divalent metal transporter 1
EFSA: European Food Safety Authority
EPO: erythropoietin
FDA: U.S. Food and Drug Administration
FOBT: fecal occult blood test
GPx: glutathione peroxidase
GRAS: generally recognized as safe
HDL: high‐density lipoprotein
IDA: iron deficiency anemia
IgM: immunoglobulin M
IL‐10: interleukin‐10
LDL: low‐density lipoprotein
MDA: malondialdehyde
PICOS: Population, Intervention, Comparison, Outcomes, Study design
PRISMA: Preferred Reporting Items for Systematic Reviews and Meta‐Analyses
RBC: red blood cell
RCT: randomized controlled trial
SCFA: short‐chain fatty acid
SOD: superoxide dismutase
TNF‐α: tumor necrosis factor‐α
WHO: World Health Organization

## 1 Introduction

Iron deficiency is a leading cause of anemia, a condition characterized by a reduced number of red blood cells (RBCs) or hemoglobin, which impairs the blood's ability to transport oxygen. Globally, anemia affects a significant portion of the population with the World Health Organization (WHO) estimating that 40% of children (aged 6–59 months), 37% of pregnant women, and 30% of non‐pregnant women (aged 15–49 years) [1].

Despite the widespread use of conventional iron supplements (e.g., ferrous sulfate, ferrous gluconate), their effectiveness is frequently compromised by gastrointestinal side effects, poor adherence, and limited bioavailability due to dietary inhibitors such as phytates, polyphenols, and calcium [2]. These limitations underscore the urgent need for alternative iron sources that are highly bioavailable, well‐tolerated, and environmentally sustainable [3].

- Microalgae have emerged as promising candidates for iron supplementation due to their high iron content, improved bioavailability, and additional metabolic benefits. Among the most extensively studied species, *Chlorella vulgaris* and *Arthrospira platensis* stand out as particularly valuable sources of iron and other essential nutrients. *Galdieria sulphuraria*, a thermophilic red alga known for its exceptional ability to accumulate iron and thrive in extreme environments, has garnered attention as a potential future option.
- *C. vulgaris* contains 419.17 mg of iron per 100 g, making it one of the richest natural sources of iron. It also contains chlorophyll, which enhances the solubility and absorption of iron in the digestive tract [4].
- *A. platensis* provides 133.57 mg of iron per 100 g and is rich in phycocyanin, a pigment‐protein complex that stimulates erythropoiesis, enhances erythropoietin (EPO) production, and facilitates iron incorporation into hemoglobin [5].
- *G. sulphuraria* is a polyextremophilic red alga that thrives in acidic, high‐temperature environments. It contains high levels of iron, an exceptional amino acid profile, and thermally stable phycocyanins, which may benefit iron metabolism and reduce oxidative stress [6].

These microalgae offer unique advantages over traditional iron sources, including improved tolerability, natural bioavailability enhancers, and additional health benefits such as antioxidant and antiinflammatory properties.

Although numerous microalgae species contain iron, only a few have been extensively studied and approved for human consumption. Species such as Nannochloropsis oceanica and Haematococcus pluvialis have promising nutritional properties but lack sufficient clinical validation for anemia treatment and are not yet widely approved for dietary supplementation [7, 8].

Conversely, C. vulgaris and A. platensis have been granted generally recognized as safe (GRAS) status by the U.S. Food and Drug Administration (FDA) and approved by the European Food Safety Authority (EFSA) for human consumption [8]. Although G. sulphuraria has not yet received full regulatory approval, recent studies indicate its safety and potential as a functional food ingredient, particularly for its high iron and protein content, bioactive compounds, and environmental resilience [6].

To illustrate the comparative advantages of these microalgae, Table 1 provides an overview of key species studied for iron supplementation, highlighting their safety, regulatory status, bioavailability, and recommended supplementation doses.

**TABLE 1:** Overview of microalgae species for iron supplementation: safety, regulatory status, bioavailability, and recommended dosages.

Microalgae	Safety and tolerability	Regulatory status	Iron bioavailability	Dosage
A. platensis	GRAS; minimal side effects (mild gastrointestinal discomfort)	FDA and EFSA approved	Human: improves hemoglobin and ferritin; Animal: enhances iron absorption and RBC production	2–5 g/day (prevention), 5–10 g/day (treatment)
C. vulgaris	Well‐tolerated; mild digestive issues	FDA and EFSA approved	Human: increases hemoglobin and ferritin; Animal: enhances iron uptake, prevents anemia	3–6 g/day (general), 6–9 g/day (treatment)
G. sulphuraria	High protein content; potential iron source; no history of adverse effects	Under review for novel food approval	High iron content; contains bioavailable phycocyanin; lacks human studies on anemia treatment	Currently unregulated; proposed as a functional food ingredient
N. oceanica	Safe, limited human studies	FDA: animal feed; EFSA: under evaluation	Human: limited data; Animal: improves hematological markers	1–3 g/day with other iron sources
H. pluvialis	Safe, mainly for astaxanthin production	FDA and EFSA approved (astaxanthin)	Human: no direct anemia evidence; Animal: antioxidant effects	Not commonly used for anemia

## 2 Methods

This systematic review follows the Preferred Reporting Items for Systematic Reviews and Meta‐Analyses (PRISMA) statement guidelines for reporting systematic reviews [9].

### 2.1 Search Strategy

A systematic and comprehensive literature search was conducted across three major electronic databases—PubMed, Scopus, and Web of Science—to identify relevant studies published between October 21, 2024, and January 18, 2025.

The search query: “((*Arthrospira platensis* OR Spirulina OR *Chlorella vulgaris* OR *Galdieria sulphuraria*) AND (anemia OR ferritin OR hemoglobin OR iron))” was used to retrieve studies examining the effects of these microalgae on iron‐related parameters, including anemia, ferritin levels, hemoglobin levels, and iron absorption.

The search terms were adapted for each database to ensure broad and precise topic coverage. The inclusion of *G. sulphuraria* in the search strategy was based on its high iron accumulation capacity and ability to thrive in extreme environments, making it a potential candidate for future iron supplementation research.

However, this systematic review's primary focus remains on *A. platensis* and *C. vulgaris* while also considering related microalgae with bioavailable iron content.

Two independent researchers conducted all stages of the systematic review process. Initially, titles and abstracts of retrieved articles underwent eligibility screening. The full texts of relevant studies were then assessed for inclusion, with discrepancies resolved through discussion and consensus. Language restrictions were applied, and only articles in English were included. The reference lists of eligible studies were screened to identify further relevant research.

### 2.2 Eligibility Criteria

The comprehensive Population, Intervention, Comparison, Outcomes, Study design (PICOS) selection criteria used to identify relevant studies are presented in Table 2.

**TABLE 2:** The study's rationale and inclusion criteria are summarized using the PICOS framework.

Criteria	Description
Population	Both human and animal studies were included. Human studies involved healthy individuals or those with mild to moderate iron deficiency. Animal models, including fish, rats, and poultry, were included to investigate preclinical effects and mechanistic insights.
Intervention	Studies assessing supplementation with *A. platensis*, *C. vulgaris*, or *G. sulphuraria*, either alone or in combination with conventional iron sources (e.g., ferrous sulfate or iron‐fortified foods). Doses ranged from 1 to 10 g/day, with intervention durations between 4 and 12 weeks. Some studies explored synergistic effects between microalgae and standard iron supplementation.
Comparison	Control groups included placebo, no intervention, or standard iron supplementation (e.g., ferrous sulfate, iron‐fortified foods, or synthetic iron chelates). In animal studies, controls consisted of standard diets without additional iron supplementation.
Outcomes	The primary outcomes assessed included iron biomarkers (e.g., hemoglobin levels, serum ferritin, and iron absorption), as well as oxidative stress markers (e.g., superoxide dismutase [SOD], glutathione peroxidase [GPx]) and immune function indicators (e.g., immunoglobulin M [IgM], cytokines such as interleukin‐10 [IL‐10], and tumor necrosis factor‐α [TNF‐α]). Studies reported that *A. platensis* supplementation increased hemoglobin levels by 5%–15% and ferritin levels by 10%–25% [10]. *C. vulgaris* supplementation exhibited strong antioxidant properties, improving SOD and GPx levels, while *A. platensis* demonstrated cardioprotective benefits in beta‐thalassemia patients. Some studies indicated a synergistic effect between *A. platensis* and conventional iron sulfate supplementation, increasing iron absorption rates by 25%. Additional outcomes included reductions in lipid peroxidation and protein carbonylation and improvements in immune function.
Study design	Randomized controlled trials (RCTs) in human populations, applying either a crossover or parallel trial design, with a minimum duration of 2 weeks. In vivo animal studies were included to investigate mechanistic effects and preclinical insights. Observational studies, in vitro research, and studies lacking anemia‐related biomarkers were excluded.

Studies were excluded if they (i) lacked a control group; (ii) did not utilize an appropriate placebo/control that resembled the intervention but did not contain *A. platensis* (Spirulina) and/or *C. vulgaris*; (iii) employed a non‐standardized supplement or mixed‐source iron supplementation; and (iv) was not randomized and/or had a study duration of fewer than 2 weeks.

### 2.3 Data Extraction

Initially, two authors independently reviewed the titles and abstracts of studies retrieved from the selected electronic databases. Following this preliminary screening, all pertinent data were gathered and verified by authors to ensure both accuracy and consistency. The extracted data encompassed several essential elements related to study design, participant characteristics, intervention specifics, and outcome measures.

The recorded publication details comprised the first author's name, publication year, country, and study title. Key study characteristics included the study design, the number and description of study arms, the duration of washout and treatment periods, the total number of participants in both the intervention and control groups, the number of participants who completed the study, and the types and doses of supplementation with *A. platensis* (Spirulina) and/or *C. vulgaris* (e.g., capsules, powders, or enriched forms) foods.

## 3 Results

### 3.1 Literature Search and Data Extraction

**FIGURE 1:**
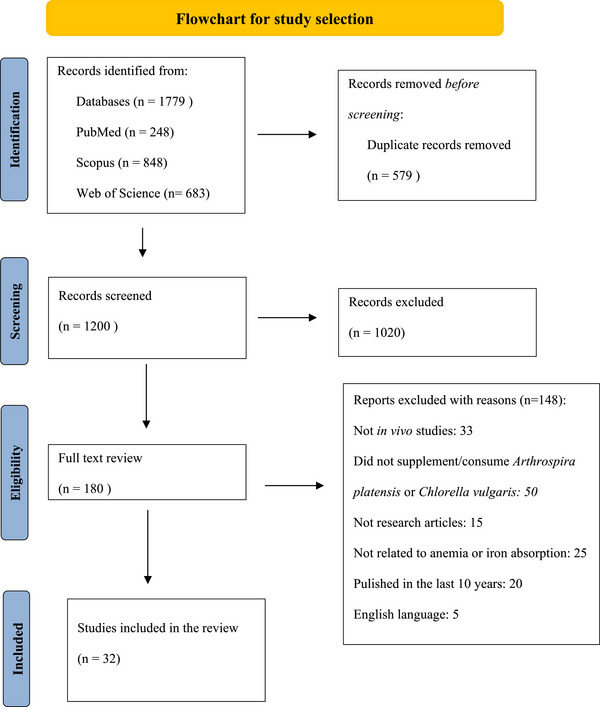
Preferred Reporting Items for Systematic Reviews and Meta‐Analyses (PRISMA) flowchart illustrating the selection process for relevant studies in this systematic review [11].

Figure 1 illustrates the study selection process according to PRISMA guidelines. A total of 1779 records were initially gathered from three major databases: PubMed (*n* = 248), Scopus (*n* = 848), and Web of Science (*n* = 683). Following the removal of 579 duplicate records, 1200 studies were left for the title and abstract screening phase.

During the screening process, 1020 studies were excluded because they did not meet the eligibility criteria. The most common reasons for exclusion were a lack of relevance to anemia or iron metabolism, studies that did not supplement or investigate *A. platensis* or *C. vulgaris*, and the inclusion of in vitro methodologies, which did not align with the review's focus on in vivo effects.

Following this, 180 full‐text articles were assessed for eligibility. A total of 147 studies were further excluded based on specific criteria. Among these, 32 studies did not involve in vivo models, making them ineligible for this review. Furthermore, 50 studies did not examine or supplement participants with *A. platensis* or *C. vulgaris*, thus failing to meet the inclusion criteria. Fifteen studies were not primary research articles; instead, they consisted of reviews, editorials, or conference abstracts, which do not present original findings. Another 26 studies were excluded because they lacked a clear connection to anemia or iron metabolism, while 20 studies were removed for being published outside the predefined time frame of 2015–2025. Lastly, five studies were excluded because they were published in languages other than English, which would have limited accessibility and standardization in data extraction.

Ultimately, 32 studies met the eligibility criteria and were included in this systematic review. These selected studies provided relevant and high‐quality data on the effects of *A. platensis* and *C. vulgaris* supplementation on iron biomarkers, oxidative stress, and immune function. This rigorous selection process ensured that the final dataset consisted solely of methodologically sound studies that directly contributed to understanding microalgae as a potential source of bioavailable iron.

Table 3 provides an overview of the 32 studies included in this systematic review. Most studies (25 studies) evaluated the effects of *A. platensis* (Spirulina) supplementation, either alone or in combination with other bioactive compounds, including *C. vulgaris*, tomato puree, lycopene, citrus lemon oil, and *Saccharomyces cerevisiae*. Moreover, five studies investigated *C. vulgaris* supplementation, while two studies assessed the combined effects of both *A. platensis* and *C. vulgaris*.

**TABLE 3:** Characteristics of the included studies evaluating the effects of *A. platensis* and *C. vulgaris* supplementation on hematological, immunological, and physiological outcomes across various populations and study designs.

Study (year)	Country	Study model	Study design	Participants	Type of intervention	Intervention/d	Control	Duration	Measured outcomes
[12]	India	Animal	Experimental study on mice	Swiss albino mice	Dietary supplementation	Spirulina, tomato puree, lycopene	Standard feed control	45 days	Hematological parameters, RBC count, hemoglobin levels
[13]	Egypt	Animal	Experimental study on fish	405 *Nile tilapia*	Dietary supplementation	Spirulina (1 g/100 g diet), *S. cerevisiae* (4 g/kg diet)	Standard feed control	Not specified	Growth, hematology, biochemistry, gene expression, histopathology
[14]	Egypt	Human	Interventional study	90 children (60 thalassemia, 30 healthy controls)	Dietary supplementation	Spirulina, 250 mg/kg/day	Healthy control group	3 months	Hemoglobin levels, transfusion frequency, cardiac function
[15]	Egypt	Animal	Experimental study on fish	*Nile tilapia*	Dietary supplementation	*C. vulgaris*, 50 g/kg diet	Standard feed control	6 weeks	Antioxidant enzyme activity, mortality rate, pesticide toxicity
[16]	Egypt	Animal	Experimental study on fish	Male *Nile tilapia*	Dietary supplementation	*A. platensis*, 1 g/kg diet + citrus limon oil	Standard feed control	2 months	Growth performance, immunity, antioxidant activity
[17]	India	Animal	Experimental study on mice	Swiss albino mice	Dietary supplementation	Spirulina, Tamarind fruit pulp (230 mg/kg body weight)	Standard feed control	7 and 90 days	Erythrocyte, leukocyte, platelet counts, hemoglobin levels
[18]	Egypt	Animal	Experimental study on fish	400 *Nile tilapia* fingerlings	Dietary supplementation	*C. vulgaris* extract with magnetic iron nanoparticles	Standard feed control	90 days	Immune response, infection severity, antioxidant function
[19]	Pakistan	Animal	Experimental study on poultry	180 broiler chickens	Dietary supplementation	*C. vulgaris*, 0.5% and 1.0%	Standard poultry feed	42 days	Toxicopathological, hematological, biochemical changes
[10]	Egypt	Animal	Experimental study on fish	*Nile tilapia*	Dietary supplementation	Spirulina, 20 and 40 g/kg diet	Standard feed control	8 weeks	Growth, immunity, oxidative stress biomarkers, platelet count
[20]	India	Animal	Experimental study on fish	Indian knife fish (*Notopterus notopterus*)	Dietary supplementation	Spirulina supplementation (10% and 100%)	Commercial diet without Spirulina	7 and 28 days	Oxidative stress biomarkers, antioxidant defense markers, iron accumulation
[21]	Iran	Animal	Interventional study on diabetic rats	36 Streptozotocin‐induced diabetic Wistar rats	Dietary supplementation	Spirulina and *C. vulgaris* supplementation	Diabetic and healthy control groups	28 days	Hemoglobin levels, RBC, WBC, platelet count, plasma iron, selenium, glutathione peroxidase activity
[22]	China	Animal	Experimental study on anemic rats	Iron‐deficient anemic rats	Dietary supplementation	Spirulina, *C. vulgaris*, and *Synechococcus* as iron sources	Ferric citrate and FeSO4	Not specified	Hemoglobin regeneration efficiency, intestinal iron absorption
[23]	Malaysia	Animal	Experimental study on African catfish	Juvenile *Clarias gariepinus*	Fishmeal replacement	Spirulina and *C. vulgaris* diet (50%, 75%)	100% fishmeal diet	Not specified	Growth performance, antioxidant enzyme activity, hematological parameters
[24]	UK	Human	Double‐blinded randomized crossover trial	17 recreationally active cyclists	Dietary supplementation	6 g/day Spirulina	Placebo	14 days	Hemoglobin levels, VO2 max, heart rate, lactate response, time to fatigue
[25]	Iran	Animal	Experimental study on Caspian brown trout	Juvenile *Salmo trutta caspius*	Dietary supplementation	Spirulina (2%, 4%, 6%, 8%)	Standard fish diet	10 weeks	Growth performance, hematological parameters, immune response markers
[26]	Brazil	Animal	Experimental study on shrimp and fish	Pacific white shrimp and juvenile *Nile tilapia*	Dietary supplementation	Microalgae (*Scenedesmus obliquus*) addition	No microalgae	62 days	Hemato‐immunological parameters, serum protein levels, immune cell counts
[27]	Brazil	Animal	Experimental study on tambaqui	*Colossoma macropomum*	Dietary supplementation	*A. platensis* and *C. vulgaris* (5%–10%)	Standard fish diet	20 days	Hematological parameters, immune response, parasite resistance
[28]	Kenya	Human	Randomized controlled trial	240 iron‐deficient children (6–23 months)	Dietary supplementation	Spirulina corn soy blend (SCSB)	Corn soy blend (CSB) and placebo	6 months	Plasma hematocrit, recovery rate from iron deficiency anemia
[29]	Peru	Human	Systematic review and meta‐analysis	338 individuals with obesity, diabetes, or dyslipidemia	Dietary supplementation	Spirulina supplementation	Placebo	Varied	Lipid profile, glucose metabolism, anti‐inflammatory markers
[30]	Egypt	Animal	Experimental study on irradiated rats	Male albino rats exposed to gamma radiation	Dietary supplementation	Spirulina (300 mg/kg) and *C. vulgaris*	Non‐supplemented irradiated group	2 weeks (pre‐ and post‐irradiation)	Hematological, liver, kidney function, oxidative stress markers
[31]	Egypt	Animal	Experimental study on rabbits	100 growing rabbits	Dietary supplementation	*C. vulgaris* (0.5–1.5 g/kg diet)	Standard rabbit diet	Varied	Growth, carcass traits, hematology, immune response, antioxidant status
[32]	Russia	Animal	Experimental study on broiler chickens	90 broiler chickens	Dietary supplementation	Spirulina (5 g/kg feed)	Standard poultry diet	42 days	Blood morphological parameters, hematology
[33]	Iraq	Animal	Experimental study on fish	Common carp (Cyprinus carpio)	Dietary supplementation	Spirulina (10 mg/kg feed)	Pellet feed without spirulina	90 days	Growth, hematology, lipid profile, enzyme activity
[34]	Tunisia	Animal	Experimental study on rats	Female rats exposed to lead toxicity	Dietary supplementation	Spirulina (5.3 g/kg)	Lead‐exposed non‐supplemented group	4 weeks	Hematology, nephrotoxicity markers, DNA damage
[35]	India	Human	Double‐blind randomized comparative study	Young anemic women (18–21 years)	Dietary supplementation	Spirulina (3 g/day)	Placebo	90 days	Hemoglobin, serum ferritin levels
[36]	Egypt	Human	Single‐arm clinical trial	Children with beta‐thalassemia and Hepatitis C	Dietary supplementation	Spirulina	None (single‐arm study)	3 months	Blood picture, immunity markers, viral load, liver function
[37]	Cambodia	Human	Single‐blind placebo‐controlled cross‐over trial	179 preschool children	Dietary supplementation	Spirulina (2 g/day)	Placebo	5 weeks (cross‐over design)	Weight gain, height, anemia prevalence
[38]	Japan	Animal	Experimental study on mice	C57BL/6J mice exposed to lead and obesity	Dietary supplementation	Spirulina	Lead‐exposed non‐supplemented group	Not specified	Hematocrit, oxidative stress markers, adipose tissue weight, lipid profile
[39]	Iran	Human	Randomized, double‐blinded, placebo‐controlled trial	80 adults with ulcerative colitis	Dietary supplementation	Spirulina (1 g/day)	Placebo	8 weeks	Serum iron, ferritin, anemia parameters, fecal occult blood test (FOBT)
[40]	Bangladesh	Animal	Experimental study on layer chickens	40 laying hens	Dietary supplementation	Spirulina (4 g/kg feed)	Standard poultry feed	4 weeks	Hematology, lead toxicity markers, productive performance
[41]	Saudi Arabia and Egypt	Animal	Experimental study on *Nile tilapia*	180 *Nile tilapia* fry	Dietary supplementation	Spirulina (0.125–1.0 g/kg diet)	Vegetarian diet without spirulina	12 weeks	Growth performance, feed utilization, hemato‐biochemical parameters
[42]	Iran	Animal	Experimental study on fish	180 rainbow trout (*Oncorhynchus mykiss*)	Dietary supplementation	Spirulina platensis (2.5%, 5%, 7.5%, and 10%) replacing fishmeal	Standard fishmeal‐based diet (0% Spirulina)	10 weeks	RBC, WBC, hemoglobin, hematocrit, cholesterol, triglyceride, high‐density lipoprotein (HDL), low‐density lipoprotein (LDL), cortisol, glucose, total protein, albumin

Regarding study design, 25 studies were experimental, conducted on animal models, including mice, fish (*Nile tilapia*, African catfish, Caspian brown trout, tambaqui, and common carp), poultry (broiler chickens and layer hens), rabbits, and shrimp. Out of these, 17 studies specifically focused on fish, evaluating growth performance, immunity, hematology, and oxidative stress biomarkers. Four studies were clinical trials involving human participants, including children with iron deficiency anemia (IDA), beta‐thalassemia, or ulcerative colitis, as well as recreationally active cyclists and young women with anemia. The remaining studies included a systematic review and meta‐analysis on obesity, diabetes, and dyslipidemia, as well as a randomized controlled trial (RCT) evaluating *A. platensis* supplementation in preschool children.

In terms of interventions, dietary supplementation with *A. platensis* ranged from 0.125 to 6 g/day, while *C. vulgaris* supplementation was administered at 0.5% to 10% of the diet. Some studies also included iron‐enriched microalgae formulations or Spirulina in combination with nanoparticles. Control groups varied across studies, with most utilizing standard diets or placebo treatments.

The duration of supplementation varied from 7 days to 12 months. The shortest interventions were conducted in animal studies evaluating the acute effects of Spirulina on oxidative stress and hematology. In contrast, longer‐term studies focused on growth, metabolism, and disease mitigation in both animals and humans.

The measured outcomes varied by study population but essentially included hematological parameters (hemoglobin, RBC count, and hematocrit), oxidative stress biomarkers, growth performance, immune response markers, and biochemical indicators such as lipid profiles, iron status, and inflammation markers. Notably, several studies have explored the protective effects of Spirulina against toxicity induced by heavy metals, including lead, fluoride, and pesticides.

These findings provide a comprehensive understanding of the potential benefits of *A. platensis* and *C. vulgaris* supplementation, particularly in enhancing hematological health, immune function, and oxidative stress resilience across different biological models and health conditions.

### 3.2 Study Characteristics and Geographical Distribution

**FIGURE 2:**
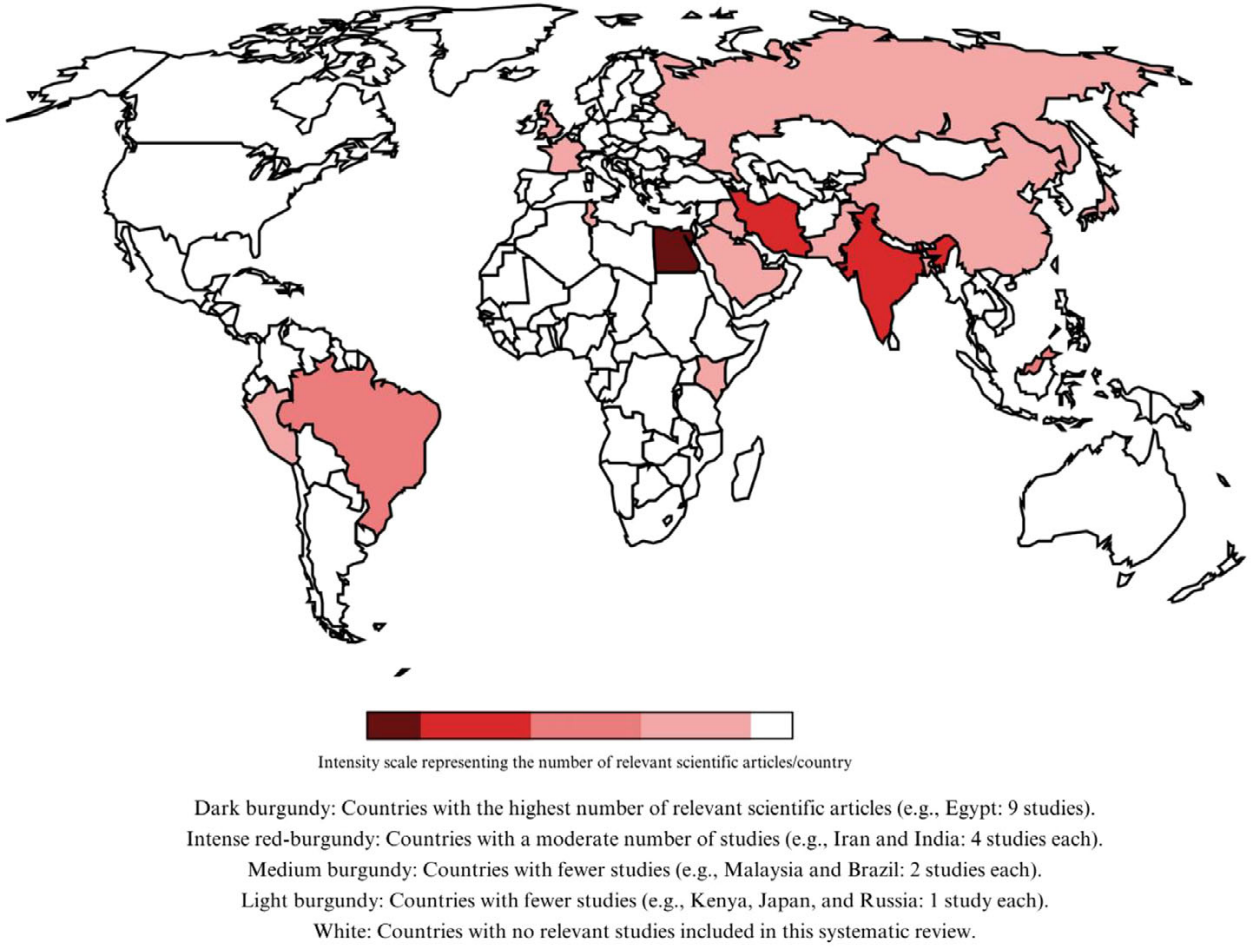
Global geographic distribution of in vivo studies on *A. platensis* and *C. vulgaris*.

Table 3 presents the characteristics of the 32 studies included in this systematic review. The geographical distribution presented in Figure 2 reveals a significant concentration of research in developing countries, with Egypt contributing the highest number of studies (*n* = 8), followed by Iran (*n* = 4) and India (*n* = 3). Other contributions came from Brazil, Malaysia, the United Kingdom, Kenya, Russia, Peru, Tunisia, Pakistan, Japan, Bangladesh, Saudi Arabia, France, Cambodia, and Iraq, each represented by one or two studies. This regional clustering reflects an increased focus on cost‐effective nutritional interventions, particularly in areas with limited access to fortified foods or conventional iron supplements. Many of these studies were driven by the high burden of IDA and an emphasis on sustainable nutrition supported by local or government research initiatives [13, 17, 28, 30, 37].

Among the included studies, seven involved human participants. At the same time, 25 were conducted using animal models, primarily in mice, rats, fish (e.g., *Nile tilapia*, rainbow trout, African catfish, and Caspian brown trout), poultry, shrimp, and rabbits. The total number of participants across all studies exceeds 2500, encompassing both humans and animals. Gender distribution was inconsistently reported in animal studies; however, some human studies specifically focused on children [14, 37], young women [35], or adults with chronic conditions [24, 39]. This diversity in study populations illustrates the broad applicability of microalgae interventions.

Regarding methodology, most studies utilized experimental in vivo models (*n* = 25), typically assessing the physiological and hematological effects of algae under controlled conditions. Additionally, there were four RCTs, for example [24, 28, 35, 39], a crossover trial [37], a single‐arm clinical study [36], and a systematic review and meta‐analysis [29]. This methodological diversity underscores both experimental rigor and the real‐world clinical relevance of evaluating *A. platensis* and *C. vulgaris* supplementation.

The type of algae supplementation also varied. Spirulina (*A. platensis*) was the most frequently used microalga (*n* = 26), often tested alone but sometimes in combination with other compounds such as *C. vulgaris* (*n* = 6), *S. cerevisiae*, tomato puree, tamarind pulp, or iron nanoparticles [12, 18, 21, 41]. A few studies explored more advanced formulations or food‐based delivery, for example, Spirulina corn‐soy blend [28] and microalgae‐enriched poultry feed [32]. The dosage of Spirulina ranged from 0.125 g/kg to 6 g/day, while *Chlorella* was often incorporated at 0.5%–10% of dietary intake [16, 19, 31].

Study durations ranged from as short as 7 days to as long as 12 weeks, with several human trials extending up to 3 months, for example [14, 39] and one meta‐analysis integrating data across different time frames [29]. Shorter studies were often conducted in animal models to assess acute toxicity or antioxidant responses, for example [17, 20], while longer trials investigated sustained impacts on growth, immunity, or anemia recovery [24, 27].

The primary measured outcomes were consistent throughout the studies and included:

- hematological markers such as hemoglobin, RBC count, hematocrit, and platelet count [14, 22];
- iron metabolism parameters, including serum iron and ferritin [35, 39];
- oxidative stress and antioxidant markers like catalase, glutathione, and lipid peroxidation [30, 34];
- growth and feed efficiency in aquaculture and livestock species [10, 25, 42];
- immune response indicators such as leukocyte count, cytokine levels, and hemocyte density [26, 40]; and
- clinical outcomes, including anemia recovery, physical performance, and inflammatory biomarkers [24, 37, 39].

Combined, these findings provide a firm overview of microalgae‐based interventions as practical tools for enhancing hematological and immunological health. The consistent application of in vivo models, combined with an increasing number of well‐controlled human trials, underscores the translational potential of *A. platensis* and *C. vulgaris* in addressing IDA and related metabolic imbalances.

### 3.3 Safety and Tolerability

#### 3.3.1 Animal Studies

The majority of the studies evaluated safety and tolerability in animal models. Across these studies, no consistent adverse effects were reported about the consumption of either *A. platensis* or *C. vulgaris*. In a subchronic toxicity study, rats were administered dried and fresh *A. platensis* at doses of up to 1200 mg/kg body weight daily for 12 weeks. No changes were observed in body weight, organ histology, clinical chemistry, or behavior, and no mortality was reported, supporting its safety even at high doses [43].

Similarly, Damessa et al. evaluated the effects of commercially available Spirulina powder on mice, administered at 15% of their diet for 4 weeks. Biomarkers of liver function (ALT, AST), kidney function (cystatin C), and cardiac function (troponin I) showed no significant differences between the Spirulina‐fed and control groups. The concentrations of heavy metals (mercury, lead, cadmium, and arsenic) detected in the Spirulina samples were below European regulatory thresholds, confirming that contaminant levels remained within permissible safety limits in all tested specimens [44].

Yongabi Anchang et al. conducted an acute toxicity and hematological assessment of *C. vulgaris* and *A. platensis* in rats. After 6 days of exposure, animals fed 25 g/day of powdered algae showed no gross pathology or significant toxicological alterations. A single physiological modification was observed, characterized by a marked elevation in leukocyte and hemoglobin values consistent with hematopoietic stimulation, without concurrent gross or histological evidence of organ pathology or systemic toxicity [45].

Additional animal studies included in the systematic review also reported no adverse effects, even at varying doses and durations. For example, Ibrahim et al., Meshkat Roohani et al., and Abdelnour et al. observed no signs of toxicity or behavioral changes in fish and poultry models supplemented with *C. vulgaris* and *A. platensis* while noting improvements in antioxidant markers and immune function [18, 25, 31].

#### 3.3.2 Human Studies

Human trials reported similarly favorable safety profiles. In a study by El‐Shanshory and coworkers, thalassemic children received 250 mg/kg/day of Spirulina for 3 months without any documented adverse events. Clinical assessments revealed improved hemoglobin levels and a reduction in transfusion frequency, with no toxicity‐related issues or symptoms [14].

In a randomized crossover trial, Ali et al. administered 6 g/day of Spirulina to recreationally active cyclists for 14 days. No side effects were reported, and the supplement was well‐tolerated. Similarly, in a randomized, placebo‐controlled trial involving 80 adults with ulcerative colitis, Moradi et al. (2023) found that 1 g/day of Spirulina for 8 weeks did not lead to any adverse reactions, suggesting tolerability even in the chronic illness population [24, 39]. Barennes et al. conducted a placebo‐controlled crossover trial involving 179 preschool children, administering 2 g/day of Spirulina for 5 weeks. No adverse effects were reported, and the supplement was associated with positive weight gain and an improvement in anemia status. In the study by Leal‐Esteban et al., young anemic women consumed 3 g/day of Spirulina for 90 days. Once again, the intervention was well‐tolerated, with no adverse events reported throughout the supplementation period [35, 37].

Additional literature supports these findings. According to a safety review by Sotiroudis and Sotiroudis, Spirulina has a long history of safe use and lacks inherent toxicity. The United States Pharmacopeia (USP) Expert Committee assigned Spirulina a Class A safety rating, indicating no known serious risks from consumption in standard doses [46, 47].

Of the 32 studies reviewed, only one case report has suggested potential adverse effects from Spirulina, and even this finding was unconfirmed. None of the animal studies analyzed (*n* = 25) reported mortality, behavioral changes, or organ toxicity attributed to Spirulina or *C. vulgaris*. In human studies (*n* = 7), no adverse events were reported across diverse populations, including children, women, athletes, and clinical participants.

The doses in these studies ranged from 0.125 g/kg to 6 g/day, with durations varying from 5 days to 3 months, and were administered primarily in powder or capsule form. The most commonly assessed safety biomarkers included ALT, AST, creatinine, uric acid, hemoglobin, and histopathology, and the results consistently fell within normal clinical ranges.

The evidence strongly supports the safety and tolerability of both *A. platensis* and *C. vulgaris* for short—and medium‐term use across multiple species and populations. Although high‐quality long‐term human trials remain limited, current findings do not indicate significant toxicological risks, even at relatively high doses.

## 4 Discussion

This systematic review evaluated 32 in vivo studies investigating the effects of A. platensis and C. vulgaris on iron metabolism, oxidative stress, and immune modulation. The results consistently demonstrated that both microalgae improve hemoglobin levels, RBC counts, serum iron and ferritin concentrations, and overall antioxidant capacity. These effects were evident across both animal and human studies, with the most notable hematological improvements occurring in models of iron deficiency or metabolic stress.

Furthermore, enhanced immune parameters and reductions in oxidative stress markers were often observed, highlighting the multifunctional role of these microalgae in promoting health and mitigating disease. The magnitude of these effects was most pronounced in studies with longer durations and higher dosages of supplementation [14, 15, 21, 39].

### 4.1 Mechanisms of Action

The hematinic effects of *A. platensis* are primarily attributed to its high iron bioavailability and the presence of phycocyanin, a pigment–protein complex that stimulates EPO expression, enhances iron incorporation into hemoglobin and protects erythrocytes from oxidative damage.

The amino acid profile and iron‐binding proteins enhance iron uptake at the intestinal level, promoting erythropoiesis in both healthy and anemic individuals. In contrast, *C. vulgaris* primarily exerts its effects by boosting non‐heme iron absorption, supported by its high vitamin C content, which converts ferric iron to ferrous iron. It may also downregulate hepcidin, a key regulator of iron metabolism, thereby facilitating ferroportin‐mediated iron export from enterocytes into the bloodstream [48, 49, 50].

### 4.2 Antioxidant Effects and Immune Modulation

Iron dysregulation contributes to oxidative stress, subsequently impacting erythropoiesis and iron utilization. Both *A. platensis* and *C. vulgaris* significantly enhanced antioxidant defense in vivo. Supplementation resulted in increases in antioxidant enzymes, such as superoxide dismutase (SOD), glutathione peroxidase (GPx), and catalase, while lipid peroxidation products, like malondialdehyde (MDA), were notably reduced. These effects protect erythrocyte membranes from oxidative damage and enhance iron utilization within tissues [20, 31].

Microalgal supplementation improved immune markers, such as leukocyte counts and cytokine responses, particularly in stressed models like fish exposed to pesticides or individuals with chronic inflammation. These antioxidant and immunomodulatory benefits may indirectly promote better iron status by lowering inflammation‐induced hepcidin expression [19, 34].

### 4.3 Comparative Outcomes: Arthrospira platensis versus Chlorella vulgaris

Although both species improved iron metabolism, A. platensis had a significantly greater impact on hemoglobin regeneration, RBC counts, and hematocrit. These outcomes are likely due to its high phycocyanin content and iron‐binding proteins that facilitate erythropoiesis. This effect was particularly evident in human studies involving anemic children, where supplementation with Spirulina significantly increased hemoglobin levels and reduced the need for blood transfusions [22, 24].

Conversely, C. vulgaris showed more substantial antioxidant effects, particularly in models of toxin exposure, metabolic stress, and inflammation. Its phytochemical profile—rich in polyphenols, chlorophyll, and vitamin C—contributes to these effects, making it more suitable in conditions where oxidative stress or immune dysregulation is predominant. Studies combining both microalgae have noted synergistic outcomes, particularly in models where hematological and oxidative stress parameters are simultaneously challenged [15, 21].

### 4.4 Heterogeneity and Population Context

Despite the diversity in species, dosage, study duration, and endpoints, the review revealed a consistent pattern of benefits from microalgae supplementation. Most studies have involved animal models—mainly fish—providing valuable insights into hematological and antioxidant responses, particularly in aquaculture. However, including seven human studies enhances translational validity. Outcomes were generally more pronounced in studies with longer durations (≥8 weeks) and moderate‐to‐high dosages (≥3 g/day in humans; ≥1 g/kg in animals). Subgroup analyses suggested more significant hematological improvements in children, females, and individuals with iron‐deficiency conditions. Nevertheless, few studies stratified by gender or reported long‐term follow‐up, which limits generalizability across populations [14, 35, 37, 39].

### 4.5 Clinical and Public Health Relevance

Microalgae present an attractive alternative or complement to traditional iron supplements, particularly in resource‐limited settings or among populations with high iron needs. Unlike synthetic iron salts, which often lead to gastrointestinal side effects and adherence challenges, microalgae provide a nutrient‐rich, bioavailable, and well‐tolerated solution. Their additional antioxidant, immunological, and metabolic benefits make them especially relevant for children, pregnant women, the elderly, athletes, and individuals with chronic illnesses. Furthermore, incorporating microalgae into functional foods, fortified meals, or therapeutic protocols could offer a sustainable approach to tackling global iron deficiency and malnutrition [4, 28, 51, 52].

## 5 Limitations of the Study

Despite the promising findings of this systematic review, several limitations must be acknowledged to contextualize the strength and applicability of the evidence. Most notably, the lack of long‐term clinical studies represents a significant gap. Of the 32 studies analyzed, only seven (22%) involved human participants, while the others were based on animal models. Although preclinical studies provide valuable mechanistic insights, interspecies differences in metabolism, gastrointestinal physiology, and microbiota composition restrict the extrapolation of these findings to human populations [15, 22]. This imbalance underscores the need for more human‐centered trials with rigorous clinical design endpoints.

In addition, the human studies included in this review were generally limited in duration (ranging from 4 to 12 weeks), sample size, and geographical scope. Most trials were conducted in low‐ and middle‐income countries (e.g., Egypt, Iran, India, Kenya, and Peru), which may not accurately represent the dietary patterns, genetic diversity, and healthcare systems of higher‐income populations. Moreover, the underrepresentation of high‐income countries in the human studies included could limit how widely the results can be applied. Differences in dietary habits, access to fortified foods, and regulatory rules on supplementation vary considerably between low‐ and middle‐income countries and high‐income nations. Future research needs to specifically examine these contextual differences to ensure the findings are relevant across various socioeconomic and nutritional contexts. Another important consideration is that none of the included human studies followed participants long enough to evaluate sustained efficacy or potential cumulative toxicity of microalgal supplementation [14, 35, 39].

Although the safety profile of *A. platensis* and *C. vulgaris* seems favorable, most trials have provided only short‐term data, with mild gastrointestinal discomfort being the sole reported adverse event. The absence of long‐term safety monitoring restricts the confidence that these supplements can be recommended for chronic use, especially in vulnerable populations such as children, pregnant women, or individuals with chronic diseases [24, 37].

There is a lack of consistency in study designs throughout the literature. Considerable heterogeneity is evident regarding microalgae strains, dosage regimens, intervention durations, delivery methods, and biomarker assessments. This variability complicates direct comparisons and limits the ability to perform pooled meta‐analyses or draw consistent conclusions. The lack of standardized outcome measures—especially iron status biomarkers such as serum ferritin, transferrin saturation, hepcidin, and soluble transferrin receptors—further complicates interpretation [10, 21, 27].

## 6 Future Directions

To address these gaps, future research should prioritize well‐designed, long‐term RCTs that evaluate the clinical efficacy, tolerability, and safety of *A. platensis* and *C. vulgaris* across diverse human populations. These trials must assess long‐term outcomes beyond 12 weeks to investigate sustained effects on iron status, erythropoiesis, antioxidant defense, and metabolic regulation. Likewise, such studies would assist in detecting delayed adverse events or cumulative toxicity, particularly in populations with high iron requirements or comorbid conditions [14, 39].

Another key area involves investigating cosupplementation strategies. Although several studies suggest that *A. platensis* and *C. vulgaris* enhance iron biomarkers, the interaction between these microalgae and standard iron therapies (e.g., ferrous sulfate, iron bis‐glycinate) or synergistic micronutrients, such as vitamin C, folate, and B12, remains unclear. Future research should investigate whether combining microalgae with these agents enhances bioavailability, reduces gastrointestinal side effects, or improves hematologic recovery more effectively than either intervention alone [49, 53].

Population‐specific studies are urgently needed. Although most existing trials target anemic or otherwise healthy individuals, underrepresented groups such as pregnant women, the elderly, and patients with chronic inflammatory or metabolic diseases (e.g., diabetes, kidney disease) require focused investigation. These populations often exhibit altered iron metabolism due to hormonal, microbiome, or inflammatory changes, which may affect the efficacy and safety of microalgal supplementation [34, 48].

To enhance the interpretability and reproducibility of future studies, there is a pressing need to adopt standardized protocols. This includes uniform dosing schedules, clearly defined intervention durations, validated dietary intake assessments, and harmonized biomarker panels for iron status and oxidative stress. Researchers should also strive to control potential confounders such as dietary iron intake, inflammation, and gut microbiota composition [19, 31].

Mechanistic studies should investigate the molecular pathways through which microalgae affect iron metabolism. Although some evidence suggests that *A. platensis* and *C. vulgaris* influence key regulators such as divalent metal transporter 1 (DMT1), ferroportin, and hepcidin, the specific interactions remain poorly characterized. Additionally, the role of microbiota‐derived metabolites, such as short‐chain fatty acids (SCFAs) and siderophores, in influencing mineral absorption warrants investigation through omics‐based approaches and gnotobiotic models [53, 54].

Lastly, future efforts must aim for more significant global equity in research. Although promising data have emerged from countries such as Egypt, India, and Iran, regions with the highest burdens of iron deficiency—including Africa, parts of Southeast Asia, and Latin America—remain underrepresented in the literature. Community‐based participatory research and culturally tailored interventions may enhance acceptability and impact in these settings. Moreover, collaborations among academic institutions, public health bodies, and local stakeholders are crucial for scaling sustainable, microalgae‐based nutritional strategies worldwide [28, 55].

## 7 Conclusions

This systematic review provides compelling evidence that *A. platensis* and *C. vulgaris* benefit iron metabolism, antioxidant defense, and immune function in both animal and human models. Across diverse populations and experimental conditions, these microalgae consistently improved hematological parameters—particularly hemoglobin concentration, RBC count, and serum ferritin—while enhancing antioxidant enzyme activity and reducing oxidative stress. Well‐characterized mechanisms, including improved iron bioavailability, modulation of key regulators such as hepcidin and ferroportin, and interactions with the gut microbiota, support the biological plausibility of these outcomes.

Although *A. platensis* demonstrated greater efficacy in stimulating erythropoiesis, *C. vulgaris* excelled in enhancing oxidative and immunological biomarkers, suggesting complementary roles that may be further optimized through combined supplementation strategies. However, the current evidence base is constrained by the predominance of short‐term animal studies, regional research concentration, and variability in study design.

To translate these findings into clinically actionable insights, future research must prioritize long‐term, standardized, and population‐specific human trials that examine safety, efficacy, and implementation feasibility. Given the global burden of IDA and the urgent need for sustainable nutritional interventions, *A. platensis* and *C. vulgaris* represent promising, food‐based solutions that deserve greater scientific and policy attention. Besides their nutritional value, these microalgae provide significant environmental benefits—including low land and water use, fast growth, and ability to sequester carbon—making them appealing iron sources within sustainable food systems.

By bridging the gap between mechanistic insights and real‐world application, these microalgae can potentially transform public health nutrition, particularly in vulnerable and resource‐constrained populations.

## Conflicts of Interest

The authors declare no conflicts of interest.

## Data Availability

Data sharing is not applicable to this article, as no new data were created or analyzed in this study.
